# Risk Factors and Clinical Outcomes of Osteotomy Plane Violation by D-Hole Screws in Medial Open Wedge High Tibial Osteotomy: A Simulation and Comparative Study

**DOI:** 10.3390/medicina59122104

**Published:** 2023-11-30

**Authors:** Sung Eun Kim, Myung Ho Lee, Chan Hee Cho, Jung-In Lee, Hyuk-Soo Han, Myung Chul Lee, Du Hyun Ro

**Affiliations:** 1 Department of Orthopaedic Surgery, Seoul National University Hospital, 101 Daehak-ro, Jongno-gu, 110-744, Seoul 03080, Republic of Korea; paxgospel@gmail.com (S.E.K.);; 2 Department of Orthopaedic Surgery, Seoul National University College of Medicine, Seoul 03080, Republic of Korea; 3 CONNECTEVE Co., Ltd., Seoul 06249, Republic of Korea

**Keywords:** high tibial osteotomy, D-hole violation, risk prediction, individualized plate

## Abstract

*Background and Objectives:* Stable fixation is essential for successful healing after medial open wedge high tibial osteotomy (MOWHTO) to minimize the risk of non-union and correction loss. In Asians, potential complications such as D-hole screw osteotomy plane violation (D-hole violation) and inadequate plate fitting arise due to improper plate size. This study aimed to evaluate the risk factors for D-hole violation and compare the conventional anatomic (CA) plate with an individualized anatomic (IA) plate in MOWHTO procedures. *Materials and Methods:* A simulation study on D-hole violation using the CA plate was conducted, involving preoperative radiographs and CT scans of 64 lower extremities from 47 MOWHTO patients. Additionally, a randomized controlled study compared CA and IA plates in MOWHTO procedures with 34 patients (17 in the CA plate group; 18 in the IA plate group). Patient demographics, patient-reported outcome measures (PROMs), and radiological measures were analyzed. *Results:* In the simulation study, the rates of D-hole violation ranged from 20.3% to 59.4%, with an increase observed as the plate was distalized from 5 mm to 10 mm away from the joint line. Short stature was identified as an independent risk factor for D-hole violation (*p* < 0.001), with a cutoff value of 155.3 cm. In the randomized controlled study, no significant difference in PROMs and D-hole violation was observed between the CA plate and IA plate groups. However, the IA plate group showed better plate fitting compared to the CA plate group (*p* = 0.041). *Conclusions:* This study identified a high risk of D-hole screw osteotomy plane violations in MOWHTO procedures, particularly when the plate is positioned more distally and in individuals with a stature below 155.3 cm. It also revealed that individualized plates provide better tibial fitting compared to conventional anatomic plates, particularly in Asian populations where tibial morphology tends to be shorter than in Western populations. Therefore, evaluating patient stature and selecting tailored plates are essential to optimize plate positioning and minimize plate-related complications in MOWHTO procedures.

## 1. Introduction

High tibial osteotomy (HTO) is a well-established treatment for patients with medial compartment osteoarthritis combined with varus alignment [1,2]. During medial open wedge HTO (MOWHTO), achieving stable fixation is crucial for bone healing [3,4,5]. Various fixation systems have been introduced for MOWHTO, including short and long plates, locking and unlocking plates, and plates with or without a metal block to achieve optimal stability [6,7]. Long plates with locking screws have demonstrated superior biomechanical properties and excellent clinical outcomes [8,9]. However, complications associated with conventional anatomical (CA) plates, such as plate irritation, have been reported [10,11].

**Figure 1:**
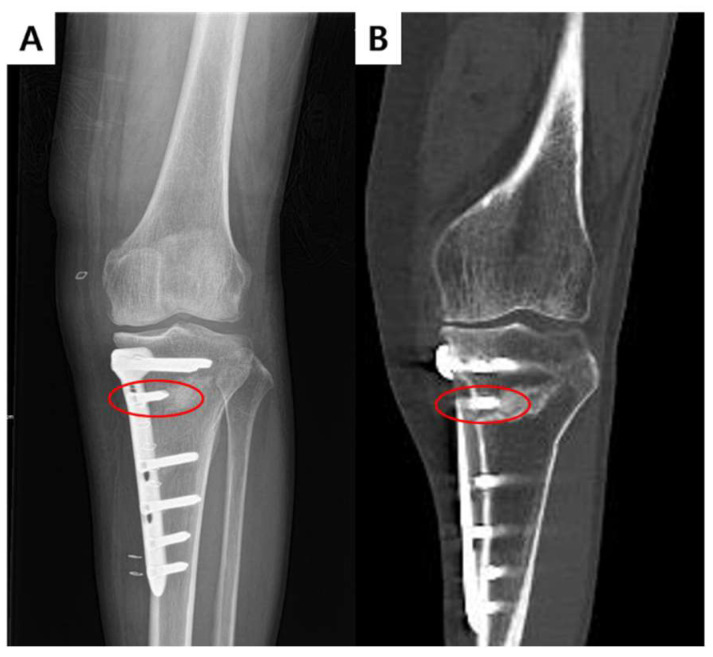
(**A**) Postoperative X-ray shows the D-hole screw of the conventional anatomic plate violating the osteotomy plane. (**B**) Postoperative 3D computed tomography scan showing D-hole violation.

The D-hole, located at the most distal screw hole of the proximal fixation part (Figure 1) [12,13], plays a role in minimizing fragment displacement after HTO. However, it has been identified as a critical concern due to the high stress and frequent occurrences of plate breakage reported in this area in previous studies [14,15,16]. The stability of the fixation may be compromised in cases where D-hole screw violates the osteotomy plane, elevating the risk of lateral hinge fractures [16]. Remarkably, the prevalence of osteotomy plane violation caused by D-hole screws (D-hole violation), specifically the unintended penetration of the osteotomy plane, has not yet been investigated. Therefore, it is important to investigate the prevalence and risk factors associated with D-hole violation.

In addition, considering the differences in proximal tibia morphology between Asian patients and the Western population [17,18], the CA plate can be too large or long for Asian patients, resulting in an inability to purchase the bone by D-hole screws and thereby leading to osteotomy plane violation. Thus, the use of an individualized anatomic (IA) plate with a smaller diameter proximal contour and the D-hole positioned closer to the joint may be beneficial.

Therefore, the objectives of this study were twofold: first, to identify risk factors contributing to D-hole violation via a simulation study, and second, to compare the CA plate with the IA plate in terms of their propensity for D-hole screw violation and overall plate fitting. Our hypotheses were that (1) positioning the plate more distally to the joint, combined with shorter patient stature, would increase the risk of D-hole violations, and (2) the IA plate would exhibit a lower incidence of these violations, along with improved fitting.

## 2. Materials and Methods

### 2.1. D-Hole Violation Simulation Study

This study was approved by the Institutional Review Board (IRB) of the authors’ institution (No.2007-085-1141). Radiological information of patients with medial compartment osteoarthritis and varus alignment who underwent MOWHTO between February 2011 and June 2019 were retrospectively reviewed. The exclusion criteria included the following conditions: loss of lateral compartment joint space, lateral tibial subluxation of 1 cm or more, medial tibial plateau erosion of 2–3 mm or more, knee flexion angle of less than 90 degrees, correction angle greater than 20 degrees required, inflammatory arthritis such as rheumatoid arthritis or gouty arthritis, presence of peripheral vascular disease, joint inflammation due to infection or osteonecrosis, and patients with stroke, Parkinson’s disease, or other neurological conditions. Preoperative teleradiograms and CT scans of the lower extremity were analyzed.

On the preoperative radiographs, tibial length, tibial width, medial proximal tibial angle (MPTA), and hip–knee–ankle angle (HKAA) were measured. Tibial length was defined as the distance between the center of the intercondylar eminence of the proximal tibia and the center of the inferior articular surface of the distal tibia, while tibial width was defined as the distance between the most medial point and the most lateral point of the tibial plateau. Tibial width was measured on both preoperative radiographs and CT scans, with the tibial length measured on radiographs being multiplied by the ratio of the tibial width measured on the CT scans and radiographs. Lower extremity CT scans were acquired using a 64-channel multi detector CT, with images taken while the knee was extended. Digital Imaging and Communications in Medicine (DICOM) data were exported to a Picture Archiving and Communication System (PACS), and the 3D multiplanar reconstruction function of PACS DICOM Viewer was used to reconstruct images and derive the virtual osteotomy plane.

**Figure 2:**
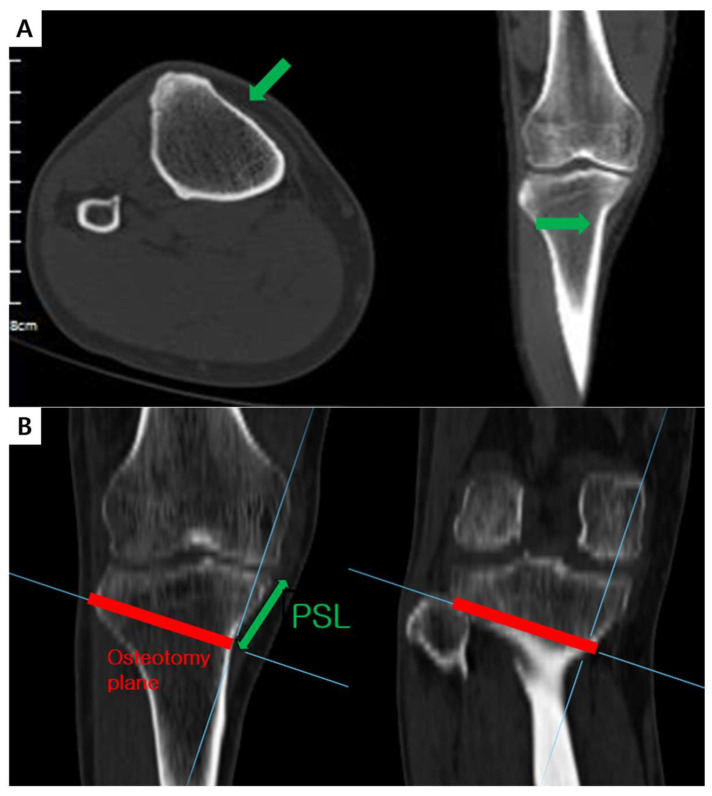
(**A**) Identification of the starting point of the osteotomy plane just above pes insertion on the 3D computed tomography scan. (**B**) Assuming the osteotomy plane from the osteotomy starting point to the proximal tibiofibular joint. After that, the proximal segment length (PSL) was measured.

The virtual osteotomy plane (VOP) was defined as an inclined line starting just above the pes insertion on the medial surface of the proximal tibia and extending toward the proximal tibiofibular junction. The proximal segment length (PSL) was defined as the distance between the starting point of the VOP and the medial corner of the tibial articular surface (Figure 2). The CA plate (TomoFix Osteotomy System; Depuy Synthes, West Chester, PA, USA) was simulated the proximal end of the plate was located 5, 7, and 10 mm distally from the articular surface of the tibial medial plateau, respectively. The distance from the proximal end of the CA plate to the upper and lower margins of the D-hole were 19 and 24 mm, respectively. When the distance between the articular surface of tibial medial plateau and the proximal end of the plate is A, the distances from the articular surface of tibial medial plateau to the upper and lower margins of the D-hole were calculated as A + 19 (B) and A + 24 (C), respectively. If the PSL was greater than C, it was defined as a non-violation. And if the PSL was between B and C, it was defined as a partial violation. If the PSL was smaller than B, it was defined as a complete violation.

### 2.2. Comparison of CA Plate and IA Plate

**Figure 3:**
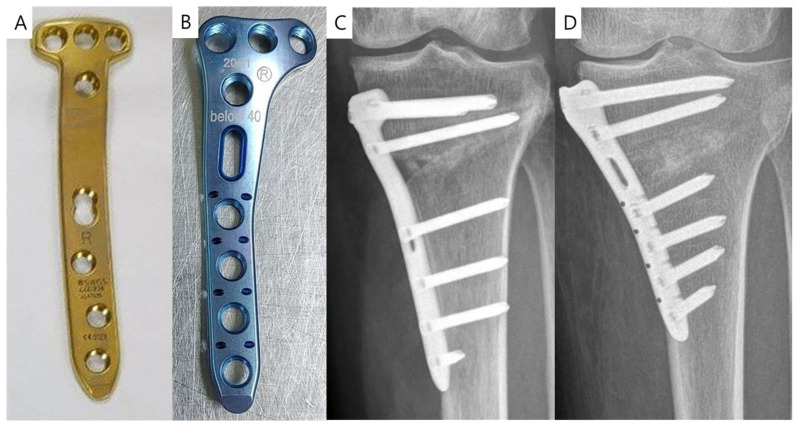
Design of the conventional anatomic (CA) plate and individualized anatomic (IA) plate. (**A**) and (**C**) show the CA plate, while (**B**) and (**D**) show the IA plate.

A randomized controlled study was performed to compare the CA plate and IA plate. This prospective study was approved by the IRB (No. 2008-105-1149) of the authors’ institution, and written informed consent was obtained from all participants. Patients with varus-aligned medial compartment osteoarthritis were enrolled between September 2020 and December 2022, using the same exclusion criteria as for the D-hole violation simulation study. The patients were randomly assigned to either the CA plate group or the IA plate (Baro-Fix HTO plate; Orthotech, Daegu, Republic of Korea) (Figure 3) group and MOWHTO were performed. The IA plate features an eccentric proximal shape, with the D-hole screw oriented more proximally compared to the CA plate. Patient demographics, preoperative and postoperative 3-month patient-reported outcome measures (PROMs) including the Knee Society Scores, and the Western Ontario and McMaster Universities Osteoarthritis Index (WOMAC) scores were collected [19,20]. Additionally, preoperative and postoperative 3-month radiological measures, such as the medial proximal tibial angle, tibial slope, and Insall–Salvati ratio [21], were evaluated. Furthermore, the fitting between the plate and the tibia was assessed by measuring the distance between the metal plate and the bone after screw fixation, and any distance exceeding 3 mm was classified as inadequate fitting [22].

### 2.3. Statistical Analysis

The sample size for the comparison between the CA and IA plates was determined based on a pilot study using Finite Element Analysis with CT scans. The statistical analysis was conducted with a significance level of α = 0.025 and a Type II error (β) of 0.20 to achieve a power of 80%. The simulation study required 54 participants, comprising 27 in each group. Categorical variables were analyzed using the chi-square or Fisher’s exact test [23], and continuous variables were analyzed using the Independent *t*-test or Analysis of Variance (ANOVA) test (for comparison between three groups). Logistic regression analysis was performed to determine the independent predictors of D-hole violation, considering results with a *p*-value of <0.05 as statistically significant. The cutoff values for characteristics capable of distinguishing between the violation and non-violation groups were determined using receiver-operating characteristic (ROC) curve analysis. All analyses were conducted using the SPSS version 25.0 software package (IBM Corp., Armonk, NY, USA). Radiological measurements were performed by two blinded investigators, and their inter-observer reliability (intra-class correlation coefficients, 0.947–0.981) and test–retest reliability (intra-class correlation coefficients, 0.962–0.986) were satisfactory.

## 3. Results

### 3.1. D-Hole Violation Simulation

A total of 64 lower extremities from 47 patients, who met the inclusion and exclusion criteria, were included in the final analysis. Table 1 presents the baseline characteristics of these patients. The rates of partial or complete violation of the VOP were 20.3%, 32.9%, and 59.4% when the proximal end of the plate was positioned at 5, 7, and 10 mm from the joint line, respectively. ANOVA showed significant differences in average height and tibial length among the three groups, but not in MPTA and HKAA (Table 2). Logistic regression analysis identified short height as an independent risk factor for D-hole violation (Table 3; odds ratio, 0.701; 95% confidence interval: 0.577–0.852; *p* < 0.001). Other variables were not significantly associated with D-hole violation.

**Figure 4:**
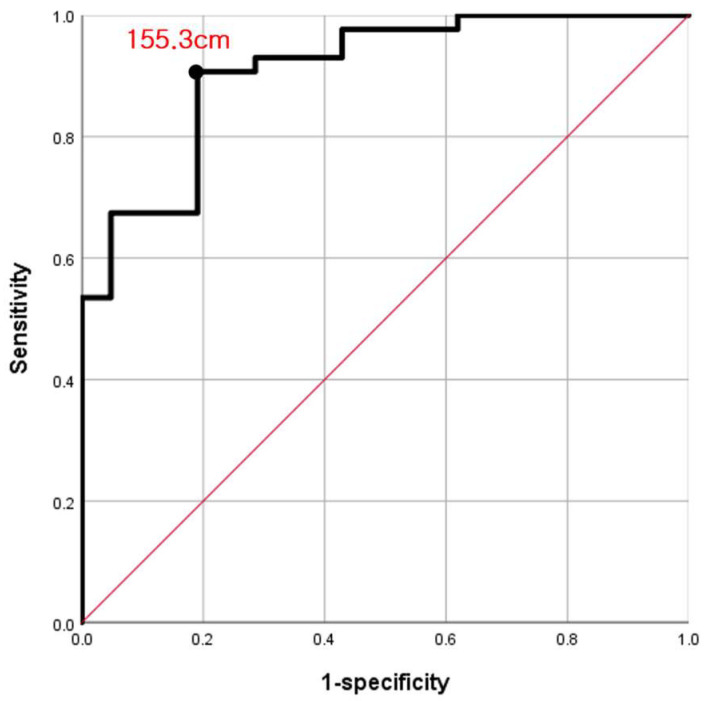
Receiver-operating characteristic (ROC) analysis. The ROC curve of height for predicting D-hole violation is shown.

ROC curve analysis was performed to determine a cutoff value for height with high sensitivity and specificity. The area under the curve for D-hole violation was 0.908 (95% confidence interval: 0.835–0.981, *p* < 0.001) (Figure 4). The optimal cutoff value for height to predict D-hole violation was found to be 155.3 cm (sensitivity, 90.7%; specificity, 81%). Therefore, a height less than 155.3 cm was associated with an increased risk of D-hole violation.

### 3.2. Comparison of CA Plate and IA Plate

**Figure 5:**
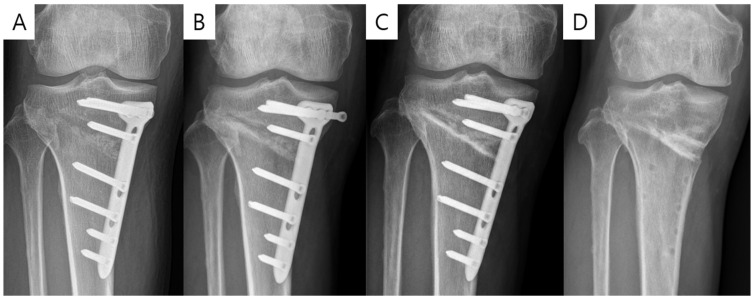
Radiography of screw loosening in a patient with conventional anatomic plate. (**A**) Immediate postoperative radiograph displaying D-hole violation. (**B**) Three-month postoperative radiograph showing proximal screw loosening and radiolucency around D-hole screw. (**C**) Radiograph following proximal screw reinsertion. (**D**) One-year postoperative radiograph.

Table 4 displays the preoperative and postoperative 3-month characteristics of the CA plate group and IA plate groups. During the study period, thirty-four patients were enrolled (17 for the CA plate group and 18 for the IA plate group). Age, body mass index, and PROMs did not exhibit any statistically significant differences between the two groups (all *p* > 0.05). Although D-hole violation was observed in 3 cases (21.4%) in the CA plate group and in 1 case (5.5%) in the IA plate group, the difference was not statistically significant (*p* = 0.338). In addition, a comparison between the D-hole violation group (*n* = 4) and the unviolated group (*n* = 30) was performed, but it did not show statistical difference in postoperative 3-month PROMs (all *p* > 0.05). Plate fitting showed a significant difference between the two groups, with the CA plate group (*n* = 6, 35.3%) showing more inadequate fitting compared to the IA plate group (*n* = 1, 5.5%) (*p* = 0.041). Three patients in the CA plate group reported skin bulging due to the plate. One patient in the CA plate group, who showed a D-hole violation, experienced proximal screw loosening and radiolucency around the D-hole screw. This patient underwent proximal screw reinsertion under local anesthesia and subsequently had hardware removal at one year postoperative without nonunion (Figure 5). During the 3-month follow-up, no complications were observed in the IA plate group, such as infections, wound complications, or fractures.

## 4. Discussion

The most significant finding of this study was the relatively high risk of D-hole violation during MOWHTO procedures using a CA plate in Asian patients. Distal placement of the plate and a shorter stature were associated with an increased risk of D-hole violation. Moreover, individualized plate designed for Asian patients demonstrated superior fitting with the tibia compared to conventional anatomic plate.

Using the D-hole screw for fixation in HTO is important for stability and reducing fragment displacement, in contrast to HTO procedures that do not use D-hole screw fixation [13]. However, the stability of the fixation may be compromised in cases where D-hole screw violates the osteotomy plane, elevating the risk of lateral hinge fractures [14,15,16]. In this study, the osteotomy plane violation rates ranged from 20.3% to 59.4%, with the highest rate of 59.4% observed when the (CA) plate was positioned 1 cm below the joint line. This finding is consistent with those from a previous study [24]. These violation rates increased as the distance from the articular surface of the medial tibial plateau to the proximal end of the CA plate increased. The assessment of D-hole violations was based on the position of the pes insertion, situated just below the osteotomy starting point. Although the anatomical location of the pes insertion is well documented [25], the exact distance from the medial tibial plateau to the proximal border of the pes insertion remains less defined in the literature [25,26]. A cadaveric study revealed a mean distance of 42 ± 7 mm between the ventral margin of the medial tibial plateau and the proximal limit of the conjoined tendon [27]. In the present study, we estimated the PSL, defined as the distance between the anteromedial margin of the medial tibial plateau and the proximal limit of the conjoined tendon, and found an average PSL of 32.9 ± 4.4 mm. Notably, the mean height of subjects in this study (160 cm) was lower compared to previous studies (mean 168 cm). Furthermore, a height less than 155.3 cm was associated with a higher risk of D-hole violation. Therefore, special attention is required when distalizing the plate and performing MOWHTO in individuals of shorter stature.

In this study, the difference in D-hole violation rate was larger in the CA plate group (21.4%) compared to the IA plate group (5.5%), and the IA plate exhibited better fitting compared to the CA plate. Previous studies have explored the optimal size and design of HTO plates [28,29], but there is limited research on which plate is suitable for different populations. In the Asian population, with their smaller height and tibia size [17], using an individualized plate that conforms to the patient’s body shape may lead to better fitting post-surgery and avoid complications such as bulging and plate irritation.

This study had several limitations. First, the simulation study used an image-processing technique, which may not fully represent real surgical values. If the D-hole screw is likely to violate the osteotomy plane during actual surgery, alternative methods, such as repositioning the plate or distalizing the osteotomy plane, may be employed to prevent D-hole violations. Second, D-hole violations were defined based on the screw head position in our study. In real surgery, the D-hole screw could penetrate the middle point of the osteotomy plane, reducing the holding force in the lowest portion of the “safe zone” [30]. Further investigations considering this aspect are required. Third, the sample size in the randomized controlled study was relatively small, potentially introducing bias. Moreover, this study did not have a long-term follow-up, and the results of PROMs according to D-hole violation may differ over time. However, the chosen follow-up duration was strategically selected to align with our study’s main goals, focusing on the D-hole screw violations and the fitting comparison of IA and CA plates, which were adequately captured within this timeframe.

## 5. Conclusions

This study identified a high risk of D-hole screw osteotomy plane violations in MOWHTO procedures, particularly when the plate is positioned more distally and in individuals with a stature below 155.3 cm. It also revealed that individualized plates provide better tibial fitting compared to conventional anatomic plates, particularly in Asian populations where tibial morphology tends to be shorter than in Western populations. Therefore, evaluating patient stature and selecting tailored plates are essential to optimize plate positioning and minimize plate-related complications in MOWHTO procedures.

## Figures and Tables

**Table 1:** Baseline characteristics of patients included in the D-hole violation simulation study.

47 Patients (64 Knees)	
Age (years)	53.1 ± 10.3
Sex (male/female) ratio	0.39 (18/46)
Height (cm)	160.1 ± 9.9
Weight (kg)	67.3 ± 11.4
Body mass index (kg/m^2^)	26.2 ± 3.7
Tibial length (mm)	314.7 ± 30.3
Tibial width (mm)	69.3 ± 5.4
Medial proximal tibial angle (°)	83.3 ± 3.1
Hip–knee–ankle angle (°)	8.5 ± 2.6
Proximal segment length (mm)	32.9 ± 4.4

Values are presented in means ± standard deviations.

**Table 2:** Patient characteristics of the simulation study grouped by distance from articular surface to proximal end of conventional anatomic plate and D-hole violation.

Distance	5 mm			7 mm			10 mm		
Violation	NV	PV	CV	NV	PV	CV	NV	PV	CV
Prevalence (%)	79.7	15.6	4.7	67.2	26.6	6.3	40.6	35.9	23.4
Height (cm)	162.8	151.9	141.3	164.4	152.9	144.4	168.5	157.4	150
	*p* < 0.001			*p* < 0.001			*p* < 0.001		
Tibial length (mm)	319.6	302.6	273	322.6	299.2	285	330.3	309.9	295.1
	*p* = 0.012			*p* = 0.002			*p* = 0.001		
Medial proximal tibial angle (°)	83.2	82.9	85.8	82.7	83.8	86.3	82.8	83.3	83.9
	n.s.			n.s.			n.s.		
Hip-knee-ankle angle (°)	8.4	9.5	6.8	8.7	8.2	7.8	8.4	8.6	8.4
	n.s.			n.s.			n.s.		

NV, non-violation; PV, partial violation; CV, complete violation; n.s., not significant.

**Table 3:** Risk factors of D-hole violation in the simulation study.

	OR (C.I.)	*p*-Value
Height (cm)	0.701 (0.577–0.852)	<0.001
Weight (kg)	-	n.s.
Body mass index (kg/m^2^)	-	n.s.
Tibial length (mm)	-	n.s.
Tibial width (mm)	-	n.s.
Medial proximal tibial angle (°)	-	n.s.
Hip knee ankle angle (°)	-	n.s.

OR, odds ratio; C.I., 95% confidence interval; n.s., not significant.

**Table 4:** The preoperative and postoperative 3 months characteristics of the CA plate group and IA plate groups.

	CA Plate (*n* = 17)	IA Plate (*n* = 18)	*p*-Value
Age (years)	54.2 ± 5.8	58.3 ± 4.2	0.087
Body mass index (kg/m^2^)	25.1 ± 3.4	24.3 ± 3.2	0.647
Preop KSKS	46.1 ± 13.0	52.5 ± 13.8	0.393
Preop KSFS	44.1 ± 6.7	41.7 ± 12.0	0.243
Preop WOMAC (pain)	8.1 ± 3.3	9.5 ± 2.1	0.391
Preop WOMAC (stiffness)	4.4 ± 1.9	5.3 ± 1.2	0.307
Preop WOMAC (function)	29.4 ± 4.4	34.5 ± 5.5	0.075
Preop MPTA	86.4 ± 1.7	84.7 ± 3.2	0.257
Preop tibial slope	12.2 ± 4.0	12.5 ± 2.5	0.862
Preop Insall-Salvati ratio	1.1 ± 0.1	1.0 ± 0.2	0.807
Postop 3 m KSKS	90.1 ± 6.7	94.4 ± 3.0	0.201
Postop 3 m KSFS	72.0 ± 20.2	84.3 ± 7.4	0.154
Postop 3 m WOMAC (pain)	2.8 ± 1.8	2.9 ± 2.0	0.935
Postop 3 m WOMAC (stiffness)	1.6 ± 1.9	1.6 ± 1.3	0.985
Postop 3 m WOMAC (function)	15.9 ± 10.5	16.6 ± 9.7	0.896
Postop 3 m MPTA	93.5 ± 3.6	90.1 ± 0.9	0.152
Postop 3 m tibial slope	15.2 ± 1.9	11.8 ± 3.2	0.060
Postop 3 m Insall-Salvati ratio	1.1 ± 0.1	1.1 ± 0.2	0.867
D-hole violation (%)	3/14 (21.4%)	1/18 (5.5%)	0.338
Plate fitting (inadequate, %)	6/17 (35.3%)	1/18 (5.5%)	0.041 *

* statistically significant at *p* < 0.05. CA, conventional anatomic; IA, individualized anatomic; Preop, preoperative; Postop 3 m, postoperative 3 months; KSKS, Knee Society knee score; KSFS, Knee Society function score; WOMAC, Western Ontario and McMaster Universities Osteoarthritis Index; MPTA, medial proximal tibial angle.

## Data Availability

The data that support the findings of this study are available from the corresponding author, upon reasonable request.
